# Food Safety Knowledge, Attitude, and Practices of Restaurant Food Handlers in Jeddah City, Saudi Arabia

**DOI:** 10.3390/foods13142176

**Published:** 2024-07-10

**Authors:** Wejdan F. Alzhrani, Israa M. Shatwan

**Affiliations:** Department of Food and Nutrition, Faculty of Human Sciences and Design, King Abdulaziz University, Jeddah, Saudi Arabia; walzhrani0064@stu.kau.edu.sa

**Keywords:** knowledge, attitude, practice, food safety, food handler, age, year of experience, salary

## Abstract

Improper food handler (FH) practices in food service areas, such as restaurants, can result in foodborne diseases (FBD). To reduce FBD cases, the food safety knowledge, attitudes, and practices (KAP) of FHs working in restaurants in Jeddah City and the correlation between their sociodemographic information and KAP scores were investigated in this study. A self-administered questionnaire comprising four parts (socioeconomic information and food safety KAP) was distributed among 389 FHs. Out of the FHs surveyed, 94.1% were male and 68% were certified. The average scores were 9.3 ± 1.8, 9.3 ± 1.3, and 8.9 ± 1.1 for KAP, respectively. Most FHs (82.2%) belonged to the good KAP group, and the rest (17.7%) were in the poor KAP group. FHs aged 50 years and above (9.6 ± 0.6), those with over 10 years of working experience (9.2 ± 0.9), and are married (9.1 ± 1.1) had the highest scores for practice compared with other FHs (*p* = 0.01 for all). Salary is directly correlated with food safety attitudes (*p* = 0.04). The findings confirmed a high score for KAP among FHs working in Jeddah. Nevertheless, more focus must be given to training younger, single, and less-experienced FHs, necessitating education courses with practical and theoretical aspects. Further studies from all regions of Saudi Arabia are necessary to generalize the study findings.

## 1. Introduction

Food safety is a global concern affecting the public health of people of all ages, races, genders, and income levels [1]. Access to enough safe and nutritious food is one of the top priorities for ensuring food security among the population and promoting public health [2]. Food service practices in commercial sectors have a significant impact on food safety, as improper food handling can result in serious outbreaks of foodborne diseases [3,4]. Foodborne diseases are estimated to affect 600 million people globally and are associated with increased morbidity and mortality [5]. In 2020, the number of foodborne diseases cases in Saudi Arabia was 3081 according to the Saudi Arabia Ministry of Health Statistical Yearbook 2020 report [6]. Several factors are associated with foodborne diseases, including poor compliance with food safety rules, ineffective regulatory systems, a lack of education, and inadequate food safety laws [7,8]. Food handlers (FHs), who prepare, store, serve, or move food as part of their jobs, play an important role in preventing foodborne diseases [9]. Studies conducted in different countries demonstrated a correlation between FHs practices in food service areas, including restaurants, and foodborne diseases cases. For example, 34.6% of foodborne diseases in Taiwan [10], 56.0% in the US [11], 21.0% in China [12], and 64.5% in the Qassim region in Saudi Arabia during summer [13] were because of improper food practices in restaurants. To prevent foodborne diseases, FHs must receive sufficient education and training courses to acquire the skills and knowledge required to maintain food safety practices and concepts prior to practical experience [14,15].

Governments and federal agencies globally establish food safety policies and guidelines and initiate education programs for FHs [16,17,18,19,20]. In Saudi Arabia, the Saudi Food and Drug Authority (SFDA) sets regulations and effective monitoring procedures to ensure food safety for consumers [19]. Furthermore, the Saudi Arabia Standards Organization develops and approves Saudi standards for goods, products, and services and issues regulations for conformity assessment and certification activities [19]. With the aim of assessing compliance with these regulations, several studies were conducted to identify food safety knowledge, attitudes, and practices (KAP) among FHs in different countries [21,22,23,24,25]. Knowledge includes understanding vital information related to food handling, preparation, cooking, and storage to prevent foodborne diseases; attitude is a set of opinions and beliefs about food safety; and practice refers to performing food safety principles at work [21,26].

In view of this, the assessment of food safety KAP among FHs may contribute to a reduction in foodborne diseases among consumers. However, few studies have been conducted in Saudi Arabia to investigate KAP among FHs. A study conducted on FHs working at King Saud University restaurants and canteens reported that FHs had excellent scores on attitude and practice, the association between knowledge and training, and knowledge and attitude toward personal hygiene [26]. In two other studies conducted in hospitals, FHs working in Madinah City hospitals had good knowledge about cross-contamination, food poisoning, and food storage and high levels of food safety practices [27]. However, an earlier study in the Makka area found that hospital FHs who did not attend training courses had low levels of knowledge; in contrast, those who previously worked in hospitals implementing hazard analysis and critical control point systems had good knowledge levels [28].

There remains a gap in assessing the KAP for food safety among FHs working in food businesses in Saudi Arabia. Thus, the current study aimed to evaluate the KAP regarding food safety among FHs working in restaurants (commercial sector) in Jeddah City, the second largest city in Saudi Arabia. The commercial sector affects a large proportion of the community and, thus, the number of foodborne diseases incidents. The association between FHs sociodemographic data and KAP scores was also examined, because identifying these factors will help to improve KAP among FHs. The results of this study are intended to help prevent the spread of foodborne diseases associated with improper food KAP; providing consumers with safe food is one of the complementing elements of our sustainable future.

## 2. Materials and Methods

### 2.1. Study Design and Participants

This cross-sectional study was conducted from the 3 March to the 28 May 2023, in Jeddah City. Food handlers (FHs) working in restaurants (e.g., banquet kitchens, fast food, fine dining, and traditional food restaurants) in Jeddah City were selected as the population. A purposive and comprehensive sample was used in this study. According to Babbie (2010), a purposive or judgmental sample is a “nonrandom sample in which the units of observation are selected based on the researcher’s judgment about which ones will be most useful or representative” [29].

Because no available data on the number of FHs working in restaurants in Jeddah City exist, we used G*Power to determine the sample size needed to be able to conduct our least sensitive statistical tests at a power of 99% (α = 0.05, two-tailed). This analysis revealed that we required a sample size of at least 296 participants (effect size = 0.5, significance level = 0.05, power = 0.99). In total, 389 restaurant FHs were selected as the sample size.

The participants were chosen from the FHs visiting the Health Education Center for work in food institutions and general health, which is governed by the Jeddah Municipality. However, we were unable to select FHs attending the training classes conducted in the center from which a random sample could be drawn because the number of FHs who attended training for certification or renewal on any given day was variable. Therefore, until the required sample size was achieved, all qualified FHs present on any day chosen for data collection were included.

The inclusion criteria were restricted to FHs of both genders involved in cutting, cooking, and preparing food inside restaurant kitchens, such as chefs, head chefs, and chef assistants. For this study, only qualified FHs who directly prepared and handled potentially dangerous foods and were able to complete the self-administered questionnaire were considered. FHs who could not read were disqualified because could not take the self-administered test without assistance from readers or interviewers.

The exclusion criteria involved any management employees or employees who do not directly prepare food, including cashiers, waiters, cleaners, FHs working at university canteens, and hospital food service workers. FHs such as waiters, grocery shop attendants, and all other FHs who were not specifically engaged in distributing potentially hazardous ready-to-eat meals were also excluded.

Ethical approval was obtained from the Biomedical Ethics Research Committee of King Abdulaziz University (89-23). The participants were informed about the study’s aim and objectives and provided their electronic consent before answering the questionnaire.

### 2.2. Study Visits

FHs in Jeddah are all trained under a general program for FHs, regardless of whether they are employed in the tourism, hotel, industrial, healthcare, commercial, or education sectors. This training program lasted 3 h a day for 3 days, beginning on Tuesdays or Saturdays and ending on Thursdays or Mondays, respectively. Two training sessions were held each day: one starting at 9 a.m. and another at 1 p.m. The course ends with an exam to ensure that the FHs are certified to work at food and beverage institutions. The FHs attending these training courses for certification or recertification were asked to complete a questionnaire. FHs that received initial training were classified as untrained. The surveys were administered before the beginning of the training sessions such that responses would not be influenced by new information presented in the training program.

### 2.3. Questionnaire

A self-administered questionnaire comprising four sections was distributed among the FHs. The first section collected data on the socioeconomic characteristics of the FHs and had items for age, sex, marital status, education level, years of experience in and outside Saudi Arabia, nationality, number of food safety courses attended, number of health certificates received, monthly income (less than 500 SAR, 500–1500 SAR, 1501–2500, and more than 2500 SAR; based on salary average of FHs working in Jeddah), position in the restaurant, and type of restaurant work. The questionnaires were translated into different languages, including Arabic, English, Urdu, Hindi, Indonesian, and Filipino, according to the FHs’ primary language.

The remaining sections were developed from the Five Keys to Safer Food by the World Health Organization to assess the knowledge, attitudes, and practices (KAP) of FHs in restaurants [22,30]. Eleven structured questions on food safety knowledge were included in the second section of the questionnaire. The three possible answers were “true”, “false”, and “do not know”. The FHs’ knowledge of microorganisms, infection management, foodborne diseases, personal hygiene, and sanitary habits was specifically covered. In this study, a score of 1 was given to correct answers, whether they were true or false, and 0 was given to incorrect answers and those not known by the FHs.

The third section comprised questions about the attitudes of FHs toward food safety. This section evaluated their beliefs, opinions, values, and character traits based on 10 questions, with possible responses of “agree”, “disagree”, and “not sure”. Every reported correct attitude received a point, while all other erroneous attitudes, including “not sure”, were given a score of 0. In this study, “agree” received a point and “disagree” received 0 points.

The fourth section assessed the FHs’ cleanliness and hygiene practices. Ten constructed questions with the probable responses of “yes” and “no” were included. Every reported good cleanliness practice received a point, whereas every recorded bad hygiene practice received 0 points. This evaluation technique was applied in earlier research [22]. In this study, a response of “yes” received 1 point, and a response of “no” received 0 points.

### 2.4. Data Analysis

SPSS software (version 28) was used for statistical analysis. The data were represented in tables as frequency and percentage for the sociodemographic characteristics and answers to KAP questions and mean ± standard deviation (Std) for KAP scores. The Chi-square test was used to determine significant differences in qualitative variables. An independent *t*-test and one-way analysis of variance were used to assess the association between sociodemographic variables and KAP scores. Also, the regression model was performed to examine the association between sociodemographic variables and KAP scores. The confounders in the model were gender, age, education, monthly income, nationality, year of experience in Saudi Arabia, marital status, and number of safety courses attended depending on the testing factor. The sums of the knowledge, attitude, and practice scores were used for the total KAP score, and this total KAP score was converted to a 100% total cumulative percentage scoring. FHs with scores < 70% were classified into the poor group, whereas those with ≥70% were considered as the good group [22,31,32]. All the scores were checked for normality. Statistical significance was set at *p* < 0.05.

## 3. Results

The participants’ characteristics are listed in Table 1. The participants were predominantly male; nearly half were between 18 and 28 years old, followed by those with ages between 29 and 39 years old, and the lowest proportion of food handlers (FHs) was in the age group of 50 and above. Approximately half of the FHs were married and the other half were unmarried. A majority of the FHs possessed a high school degree or below (56.0%) and 37.3% had a diploma or bachelor’s degree. Approximately 35.0% of the FHs were paid more than 2500 SAR. Asians represented the highest nationality, followed by Africans, Saudi Arabians, and Yemeni. The majority of FHs reported that they had health certificates, whereas the others had yet to obtain a certificate. The majority of FHs had worked in Saudi Arabia for 1–5 years, with 71.7% of participants stating they had never worked outside the country. The majority of the participants were chefs and 46.3% worked in fast-food restaurants. The average knowledge, attitudes, and practices (KAP) scores were 9.3 ± 1.8, 9.3 ± 1.3, and 8.9 ± 1.1, respectively.

Table 2 presents the knowledge of food safety among FHs. The answers of FHs with good and poor knowledge were statistically different for seven statements. A total of 345 were aware that mopping with a towel could spread microbes; 21.7% of the poor group answered false to this statement compared with the 5.0% in the good group (*p* < 0.001). A relatively high proportion of FHs found it acceptable to use the same cutting board for raw and cooked food, with 30.4% in the poor group reporting the correct answer compared with 7.2% in the good group (*p* < 0.001). Although the majority of FHs answered correctly to the statement “raw food needs to be stored separately from cooked food”, 30.4% of the poor group and 3.1% of the good group chose false (*p* < 0.001). Most of the FHs in the poor group answered correctly to the statement “cooked foods do not need to be thoroughly reheated”, whereas only a quarter answered correctly for the good group (*p* < 0.001). A relatively high proportion of FHs in the poor group were aware that 40 °C was not an appropriate temperature for cooking meat compared with those in the good group (*p* < 0.001). Additionally, FHs in the poor group were aware that meat cannot be left at room temperature overnight to cool, compared with only 17.8% in the good group (*p* < 0.001). The majority of the poor group indicated that water safety cannot be identified by appearance compared with 43.1% in the good group.

Table 3 summarizes the attitudes of the FHs toward food safety and compares the good and poor groups. Answers to adherence to frequent handwashing during food preparation were statistically significant (*p* < 0.001); 31.9% of the poor group and 14.4% of the good group disagreed with frequent handwashing. The majority of FHs agreed on using meat thermometers to ensure food was cooked; however, 11.6% of the poor group disagreed compared with 4.4% in the good group (*p* = 0.003). A relatively high percentage of the poor group disagreed with thawing food in a cool place, compared with 9.7% in the good group (*p* < 0.001). Most FHs in the good group (83.1%) and 68.1% of the poor group believed that leaving cooked food out of the refrigerator was hazardous (*p* < 0.001). The answers to food inspection for freshness and edibleness between the FHs in the good and poor quality groups significantly differed (*p* = 0.01). Although most FHs agreed on the necessity of throwing foods that reached the expiry date, 8.7% of the poor group and 1.0% of the good group disagreed (*p* < 0.001).

The FH practices related to food safety and the differences between the good and poor groups are illustrated in Table 4. Food safety practices differed significantly between the FHs in the good and poor groups. FHs in the poor group did not adhere to standard food safety practices, namely, compliance with handwashing during food preparation (10.1%), cleaning of surface and equipment before re-use (10.1%), use of separate cutting boards and utensils for cooked and raw foods (18.8%), storage of raw food separately from cooked food (11.6%), use of a thermometer to check the internal temperature of meat during cooking (27.5%), reheating of food until it is piping hot (43.5%), thawing of frozen food in the refrigerator or other cool places (40.6%), refrigeration of leftovers within 2 h (46.4%), and checking of expiration date and disposing of food past their expiration (7.2%).

As demonstrated in Table 5, age (*p* = 0.01), marital status (*p* = 0.01), and years of experience (*p* = 0.01) were significantly positively associated with food safety practices, whereas salary was positively associated with food safety attitudes (*p* = 0.04). Older FHs (50 years and above) had higher practice scores than younger FHs (18–39 years). Married FHs had higher practice scores than unmarried FHs. FHs with more than 10 years of working experience had higher practice scores than FHs with less than a year of working experience. FHs with average monthly incomes of 500–1500 SAR had the highest attitude scores compared with those who earned less than 500 SAR. After adjusting for confounding factors, none of these associations remain significant. None of the FHs’ sociodemographic data were statistically correlated with knowledge or total KAP scores (*p* < 0.05). Notably, females (*p* = 0.02), Yemeni nationals (*p* = 0.04), and fast-food FH workers (*p* = 0.04) were associated with poor KAP (Table 6).

## 4. Discussion

This cross-sectional study determined the food safety knowledge, attitudes, and practices (KAP) among food handlers (FHs) in restaurants in Jeddah. The findings revealed high KAP scores among the FHs enrolled in this study. A significant difference was observed between FHs in good (82.2%) and poor (17.7%) groups in relation to some knowledge, attitudes, and practices regarding food safety. Those in the poor group were unaware that using the same towels can lead to cross-contamination and that cooked and raw food must be stored separately; however, they were aware that different cutting boards must be used for cooked and raw food; cooked food must be reheated; 40 °C is not enough for cooking meat; meat cannot be left at room temperature; and water safety cannot be identified by appearance. Additionally, the findings showed that FHs in the poor group did not adhere to handwashing, using meat thermometers, thawing food in a cool place, keeping cooked food in the refrigerator, disposing of foods that reached the expiry date, and checking the freshness and wholesomeness of the food. FHs in the poor group had low compliance with food safety practices, such as handwashing, cleaning surfaces and equipment, using separate cutting boards and utensils for cooked and raw foods, storing them separately, using a thermometer to check the temperature, reheating food, thawing frozen food in the refrigerator, refrigerating leftovers, and checking the expiration date. Moreover, the results in the poor group indicated that food safety practice scores increased as the age and years of experience increased; married FHs also had high practice scores. Attitude scores were associated with monthly income. However, none of these associations remain significant in the adjusted model. In addition, a higher percentage of females, Yemeni nationals, and fast-food FHs were in the poor KAP group.

Many FHs knew to separate raw and cooked food utensils, properly cook, and handle leftover food. This finding is comparable to those of studies conducted in Ghana, Italy, Turkey, and Kuwait [22,25,33,34]. However, this result differed from those of studies conducted in China and Pakistan, where their FHs had poor food safety knowledge and did not know about practices to reduce the risk of food contamination nor the “2 h rule” to leave food out at room temperature [23,35]. Notably, our study reported concerns about some aspects of food safety knowledge, including the use of the same towels and separate storage of cooked and raw foods. Similarly, a study conducted by Kanaan [36] indicated knowledge gaps regarding the storage of food in refrigerators and proper cleaning.

FH attitudes toward applying food safety plans are strongly associated with a decrease in the risk of foodborne diseases. A high percentage of FHs had good attitudes toward handwashing, using meat thermometers, thawing food in cool places, keeping cooked food in refrigerators, disposing of expired foods, and checking the freshness and wholesomeness of food. These findings agree with those of studies conducted in Dubai, Kuwait, and Saudi Arabia [26,37,38], but differ from those of studies conducted in Ghana and the Maldives [39,40], in which FHs had poor cleaning habits, did not know the correct refrigerator temperature [40], and were not aware that defrosted food should not be refrozen [39].

The practice score of FHs in this current study corroborates those of previous studies conducted in Madinah City, Saudi Arabia, and Dubai [27,37]. Meanwhile, the scores were higher than the practice scores reported in Bangladesh and Ethiopia [41,42]. In terms of FH compliance with good food safety practices, the vast majority of FHs reported hand washing regularly and cleaning surfaces and equipment, which is in line with studies conducted in the GCC region and the Philippines [37,38,43]. In contrast, FHs in Ghana and Bangladesh did not often practice regular handwashing and did not wash utensils such as plates, glasses, spoons, or surfaces such as tables [39,44]. Another good practice demonstrated by the FHs in our study was the separate storage of raw and cooked food using different utensils. A study conducted in southern Italy also found that 71.2% and 65.4% of FHs separated raw and cooked foods and used different utensils, plates, and chopping boards for raw and cooked foods, respectively [33].

Our study revealed a positive association between practice scores and age and years of experience, which was similar to earlier studies conducted in Ethiopia [45,46]. Older workers with more experience have an increased tendency to practice food safety standards. In contrast, a previous study conducted in Jordan confirmed a negative association between age and years of experience and practice scores, with younger FHs with fewer years of experience having higher practice scores [47]. Our study also found that practice scores were associated with marital status. This finding is supported by studies conducted in Ethiopia and the Maldives, in which single FHs have low practice scores [48].

The present study also showed that FHs who earned an average monthly income of 500–1500 SAR had the highest attitude scores compared with other FHs who earned less than 500 SAR or above 1501 SAR. In contrast with our findings, a study in Ghana did not associate monthly income with FH attitudes, but instead with FH practices [22]. One reason for this disparity might be that institutions in Saudi Arabia bear the costs of transportation, housing rent, and medical insurance, thus saving FHs from deducting these costs from their salaries. Moreover, the current study found that females, Yemeni FHs, and FHs working in fast-food restaurants demonstrated poor KAP. A Ghana study did not confirm the association between gender and KAP [22]. The number of females in our study was small; thus, we cannot draw strong conclusions based on it. Because no previous study has examined nationality or type of restaurant as factors that affect KAP scores, we were unable to compare these findings.

This study had some limitations. The cross-sectional design of the study made it difficult to draw causal conclusions. Although KAP was measured using reliable tools obtained by the WHO and has been previously used, self-reporting by FHs may introduce some social desirability, recall bias, and effect accuracy in the results. The main strength of the study was that the sample size was appropriate based on the calculations, and the findings can be generalized to other FHs working in restaurants in Jeddah.

## 5. Conclusions

Food handlers in Jeddah had high knowledge, attitudes, and practices scores, which represent higher adherence to SFDA guidelines and regulations. However, some aspects need further emphasis, such as practices leading to cross-contamination, personal hygiene (e.g., handwashing), proper methods for thawing food, and dealing with leftover food. In addition, more focus should be dedicated to improving food safety practices among single, younger, and less-experienced food handlers. Salary affects attitudes and beliefs about food safety, which needs to be considered by food service organizations. Sustained educational courses provided by authorized organizations, including practical and theoretical courses, are recommended. Further studies that include food handlers from all regions of Saudi Arabia are required to confirm our findings.

## Figures and Tables

**Table 1:** The frequencies and percentage of the demographic characteristics of food handlers and knowledge, attitudes, and practices scores.

Characterization	Total (*n* = 389) *n* (%)
Gender	
Male	366 (94.1)
Female	23 (5.9)
Age	
18–28	175 (45.0)
29–39	149 (38.3)
40–49	49 (12.6)
50 and above	16 (4.1)
Marital status	
Married	197 (50.6)
Unmarried	193 (49.4)
Educational level	
Literate	26 (6.7)
High school or below	218 (56)
Diploma or bachelor	145 (37.3)
Nationality	
Asian	144 (37.0)
African	94 (24.2)
Saudi	65 (16.7)
Yemeni	69 (17.7)
Others	17 (4.4)
Number of years of work in Saudi Arabia	
Less than 1 year	99 (25.4)
1–5 years	138 (35.5)
6–10 years	68 (17.5)
More than 10 years	84 (21.6)
Have you ever worked outside of Saudi Arabia?	
No	279 (71.7)
Yes	110 (28.3)
Your job in the restaurant	
Head chef	78 (20.1)
Chef	311 (79.9)
Do you have a health certificate?	
No	123 (31.6)
Yes	266 (68)
How many food safety courses have you attended?	
No one	87 (22.4)
One course	118 (30.3)
Two courses	82 (21.1)
More than two	102 (26.2)
Your monthly income	
Less than 500 SAR	29 (7.5)
500–1500 SAR	124 (31.9)
1501–2500 SAR	100 (25.7)
More than 2500 SAR	136 (35)
The type of restaurant you work in	
Fast food	180 (46.3)
Traditional	77 (19.8)
Fine dining	86 (22.1)
Banquet kitchen	46 (11.8)
Scores	Mean ± Std
Knowledge score	9.3 ± 1.8
Attitude score	9.3 ± 1.3
Practices score	8.9 ± 1.1

Knowledge, attitudes, and practices presented as mean and standard deviation.

**Table 2:** Knowledge about food safety among food handlers; comparison between food handlers in the good and poor groups.

Knowledge Questions	Total (*n* = 389) *n* (%)	Good (*n* = 320) *n* (%)	Poor (*n* = 69) *n* (%)
1—It is important to wash hands before handling food.			
True	385 (99.0)	316 (98.8)	69 (100.0)
False	2 (0.5)	2 (0.6)	0 (0.0)
Don’t know	2 (0.5)	2 (0.6)	0 (0.0)
*p*-Value			0.64
2—Mopping with a towel can spread microbes.			
True	345 (88.7)	285 (88.7)	54 (88.5)
False	31 (8.0)	16 (5.0)	15 (21.7)
Don’t know	13 (3.3)	13 (4.1)	0 (0.0)
*p*-Value			<0.001
3—The same cutting board can be used for raw and cooked foods.			
True	337 (86.6)	289 (90.3)	48 (69.6)
False	44 (11.3)	23 (7.2)	21 (30.4)
Don’t know	8 (2.1)	8 (2.5)	0 (0.0)
*p*-Value			<0.001
4—Raw food needs to be stored separately from cooked food.			
True	356 (91.5)	308 (96.3)	48 (69.6)
False	31 (8.0)	10 (3.1)	21 (30.4)
Don’t know	2 (0.5)	2 (0.6)	0 (0.0)
*p*-Value			<0.001
5—Cooked foods do not need to be thoroughly reheated.			
True	251 (64.5)	227 (70.9)	24 (34.8)
False	122 (31.4)	79 (24.7)	43 (62.3)
Don’t know	16 (4.1)	13 (4.0)	3 (4.9)
*p*-Value			<0.001
6—Proper cooking includes meat cooked to 40 °C.			
True	257 (66.1)	227 (70.9)	30 (43.5)
False	87 (22.4)	51 (15.9)	36 (52.2)
Don’t know	45 (11.6)	42 (13.1)	3 (4.3)
*p*-Value			<0.001
7—Cooked meat can be left at room temperature overnight to cool before refrigerating.			
True	288 (74.0)	250 (78.1)	38 (55.1)
False	88 (22.6)	57 (17.8)	31 (44.9)
Don’t know	13 (3.3)	13 (4.1)	0 (0.0)
*p*-Value			<0.001
8—Cooked food should be kept very hot before serving.			
True	338 (86.9)	280 (87.5)	58 (84.1)
False	39 (10.0)	28 (8.8)	11 (15.9)
Don’t know	12 (3.1)	12 (3.8)	0 (0.0)
*p*-Value			0.06
9—Refrigerating food only slows bacterial growth.			
True	180 (46.3)	145 (45.3)	35 (50.7)
False	183 (47.0)	151 (47.2)	32 (46.4)
Don’t know	26 (6.7)	24 (7.5)	2 (2.9)
*p*-Value			0.33
10—Safe water can be identified by the way it looks.			
True	196 (50.4)	170(53.1)	26 (37.7)
False	180 (46.3)	138 (43.1)	42 (60.9)
Don’t know	13 (3.3)	12 (3.8)	1 (1.4)
*p*-Value			0.02
11—Fruits and vegetables should be washed.			
True	384 (98.7)	316 (98.8)	68 (98.6)
False	4 (1.0)	3 (0.9)	1 (1.4)
Don’t know	1 (0.3)	1 (0.3)	0 (0.0)
*p*-Value			0.83

The chi-squared test was used to examine differences between poor and good groups; a *p*-value of 0.05 was considered statistically significant.

**Table 3:** Food handler’s answers for attitude questions; comparison between food handlers in the good and poor groups.

Attitude Questions	Total (*n* = 389) *n* (%)	Good (*n* = 320) *n* (%)	Poor (*n* = 69) *n* (%)
1—Frequent handwashing during food preparation is worth the extra time.			
Agree	313 (80.5)	267 (83.4)	46 (66.7)
Disagree	68 (17.5)	46 (14.4)	22 (31.9)
Not sure	8 (2.1)	7 (2.2)	1 (1.4)
*p*-Value			0.002
2—Keeping kitchen surfaces clean reduces the risk of illness.			
Agree	358 (92.0)	296 (92.5)	62 (89.9)
Disagree	27 (6.9)	20 (6.3)	7 (10.1)
Not sure	4 (1.0)	3 (0.9)	1 (1.4)
*p*-Value			0.34
3—Keeping raw and cooked food separate helps to prevent illness.			
Agree	378 (97.8)	313 (97.8)	65 (94.2)
Disagree	7 (1.8)	4 (1.3)	3 (4.3)
Not sure	4 (1.0)	3 (0.9)	1 (1.4)
*p*-Value			0.19
4—Using different knives and cutting boards for raw and cooked foods is worth the extra effort.			
Agree	276 (71.0)	232 (73.5)	44 (63.8)
Disagree	104 (26.7)	80 (25.0)	24 (34.8)
Not sure	9 (2.3)	8 (2.5)	1 (1.4)
*p*-Value			0.23
5—Meat thermometers are useful for ensuring food is cooked thoroughly.			
Agree	357 (91.8)	297 (92.8)	60 (87.0)
Disagree	17 (4.4)	9 (2.8)	8 (11.6)
Not sure	15 (3.9)	14 (4.4)	1 (1.4)
*p*-Value			0.003
6—Soups and stews should always be boiled to ensure safety.			
Agree	348 (89.5)	287 (89.7)	61 (88.4)
Disagree	24 (6.2)	18 (5.6)	6 (8.7)
Not sure	17 (4.4)	15 (4.7)	2 (2.9)
*p*-Value			0.52
7—Thawing food in a cool place is safer.			
Agree	313 (80.5)	269 (84.1)	44 (63.8)
Disagree	56 (14.4)	31 (9.7)	25 (36.2)
Not sure	20 (5.1)	20 (6.3)	0 (0.0)
*p*-Value			<0.001
8—I think it is unsafe to leave cooked food out of the refrigerator for more than 2 h.			
Agree	313 (80.5)	266 (83.1)	47 (68.1)
Disagree	62 (15.9)	40 (12.5)	22 (31.9)
Not sure	14 (3.6)	14 (4.4)	0 (0.0)
*p*-Value			<0.001
9—Inspecting food for freshness and wholesomeness is valuable.			
Agree	358 (92)	294 (91.9)	64 (92.8)
Disagree	12 (3.1)	7 (2.2)	5 (7.2)
Not sure	19 (4.9)	19 (5.9)	0 (0.0)
*p*-Value			0.01
10—I think it is important to throw away foods that have reached their expiry date.			
Agree	376 (96.7)	313 (97.8)	63 (91.3)
Disagree	9 (2.3)	3 (0.9)	6 (8.7)
Not sure	4 (1.0)	4 (1.3)	0 (0.0)
*p*-Value			<0.001

The chi-squared test was used to examine differences between poor and good groups; a *p*-value of 0.05 was considered statistically significant.

**Table 4:** Food handlers answers for practices questions; comparison between food handlers in the good and poor groups.

Practices Questions	Total (*n* = 389) *n* (%)	Good (*n* = 328) *n* (%)	Poor (*n* = 61) *n* (%)
1—I wash my hands before and during food preparation.			
Yes	371 (95.4)	309 (96.6)	62 (89.9)
No	18 (4.6)	11 (3.4)	7 (10.1)
*p*-Value			0.01
2—I can clean surfaces and equipment used for food preparation before re-using other food.			
Yes	371 (95.4)	309 (96.6)	62 (89.9)
No	18 (4.6)	11 (3.4)	7 (10.1)
*p*-Value			0.01
3—I use separate utensils and cutting boards when preparing raw and cooked food.			
Yes	360 (92.5)	304 (95.0)	56 (81.2)
No	29 (7.5)	16 (5.0)	13 (18.8)
*p*-Value			<0.001
4—I separate raw and cooked food during storage.			
Yes	375 (96.4)	314 (98.1)	61 (88.4)
No	14 (3.6)	6 (1.6)	8 (11.6)
*p*-Value			<0.001
5—I check that meats are cooked thoroughly by ensuring that the juices are clear or by using a thermometer.			
Yes	347 (89.2)	297 (92.8)	50 (72.5)
No	42 (10.8)	23 (7.2)	19 (27.5)
*p*-Value			<0.001
6—I reheat cooked food until it is piping hot throughout.			
Yes	304 (78.1)	265 (82.8)	39 (56.5)
No	85 (21.9)	55 (17.2)	30 (43.5)
*p*-Value			<0.001
7—I thaw frozen food in the refrigerator or other cool place.			
Yes	304 (78.1)	263(82.2)	41 (59.4)
No	85 (21.9)	57 (17.8)	28 (40.6)
*p*-Value			<0.001
8—After I have cooked a meal I store any leftovers in a cool place within 2 h.			
Yes	287 (73.8)	250 (78.1)	37 (53.6)
No	102 (26.2)	70 (21.9)	32 (46.4)
*p*-Value			<0.001
9—I check and throw away food beyond its expiry date.			
Yes	377 (96.9)	313 (97.8)	64 (92.8)
No	12 (3.1)	7 (2.2)	5 (7.2)
*p*-Value			0.02
10—I wash fruit and vegetables with safe water before eating them.			
Yes	385 (99.0)	317 (99.1)	68 (98.6)
No	4 (1.0)	3 (0.9)	1 (1.4)
*p*-Value			0.70

The chi-squared test was used to examine differences between poor and good groups; a *p*-value of 0.05 was considered statistically significant.

**Table 5:** Association between food safety knowledge, attitude, and hygiene practice scores and food handlers socio-demographic characteristics.

Sociodemographic Characteristic	Knowledge	Attitude	Practices
	Mean ± Std	Mean ± Std	Mean ± Std
Gender			
Male	9.3 ± 1.8	9.2 ± 1.3	8.9 ± 1.2
Female	9.17 ± 2.2	9.4 ± 1.5	9.1 ± 1.0
*p*-Value	0.72	0.61	0.55
Adjusted *p*-value	0.67	0.59	0.83
Age			
18–28	9.3 ± 1.8	9.4 ± 1.4	8.8 ± 1.2
29–39	9.3 ± 1.8	9.2 ± 1.2	8.8 ± 1.2
40–49	9.2 ± 1.6	9.2 ± 1.3	9.2 ± 0.9
50 and above	9.7 ± 1.8	9.6 ± 1.9	9.6 ± 0.6
*p*-Value	0.76	0.51	0.01
Adjusted *p*-value	0.67	0.60	0.06
Marital status			
Married	9.4 ± 1.7	9.3 ± 1.3	9.1 ± 1.1
Unmarried	9.2 ± 1.8	9.3 ± 1.4	8.8 ± 1.2
*p*-Value	0.35	0.98	0.01
Adjusted *p*-value	0.44	0.95	0.34
Educational level			
Literate	9.5 ± 1.8	9.4 ± 1.7	9.1 ± 0.9
High school or below	9.1 ± 1.7	9.3 ± 1.2	8.9 ± 1.2
Diploma or bachelor	9.5 ± 1.8	9.2 ± 1.3	8.9 ± 1.1
*p*-Value	0.10	0.72	0.45
Adjusted *p*-value	0.10	0.78	0.62
Nationality			
Asian	9.2 ± 1.8	9.4 ± 1.2	8.8 ± 1.2
African	9.2 ± 1.6	9.2 ± 1.2	9.0 ± 1.1
Saudi	9.7 ± 1.8	8.9 ± 1.3	9.1 ± 1.1
Yemeni	8.9 ± 1.6	9.4 ± 1.4	8.8 ± 1.1
Others	9.4 ± 2.0	9.2 ± 1.3	8.8 ± 1.1
*p*-Value	0.15	0.19	0.31
Adjusted *p*-value	0.16	0.18	0.53
Number of years of work in Saudi Arabia			
Less than 1 year	9.0 ± 1.8	9.3 ± 1.2	8.7 ± 1.3
1–5 years	9.5 ± 1.7	9.3 ± 1.4	8.9 ± 1.2
6–10 years	9.4 ± 1.6	9.3 ± 1.1	8.9 ± 1.1
More than 10 years	9.3 ± 1.9	9.3 ± 1.5	9.2 ± 0.9
*p*-Value	0.43	0.98	0.01
Adjusted *p*-value	0.31	0.99	0.13
Have you ever worked outside of Saudi Arabia?			
No	9.4 ± 1.8	9.2 ± 1.3	8.9 ± 1.1
Yes	9.2 ± 1.9	9.5 ± 1.3	8.9 ± 1.3
*p*-Value	0.45	0.09	0.53
Adjusted *p*-value	0.40	0.07	0.49
Your job in the restaurant			
Head chef	9.4 ± 2.1.7	9.2 ± 1.1	9.1 ± 1.0
Chef	9.3 ± 1.8	9.3 ± 1.4	8.9 ± 1.2
*p*-Value	0.81	0.53	0.37
Adjusted *p*-value	0.90	0.58	0.65
Do you have a health certificate?			
No	9.4 ± 1.6	9.2 ± 1.3	8.9 ± 1.1
Yes	9.3 ± 1.9	9.3 ± 1.3	8.9 ± 1.2
*p*-Value	0.73	0.32	0.65
Adjusted *p*-value	0.69	0.27	0.77
How many food safety courses have you attended?			
None	9.4 ± 1.6	9.4 ± 1.3	8.9 ± 1.1
One course	9.2 ± 1.8	9.3 ± 1.3	8.9 ± 1.2
Two courses	9.5 ± 1.9	9.2 ± 1.5	9.2 ± 0.9
More than two	9.3 ± 1.8	9.2 ± 1.2	8.9 ± 1.2
*p*-Value	0.49	0.91	0.18
Adjusted *p*-value	0.59	0.94	0.22
Your monthly income			
Less than 500 SAR	9.3 ± 1.5	9.0 ± 1.5	9.1 ± 1.1
500–1500 SAR	9.4 ± 1.9	9.6 ± 1.2	8.9 ± 1.2
1501–2500 SAR	9.1 ± 1.7	9.1 ± 1.3	8.9 ± 1.2
More than 2500 SAR	9.4 ± 1.8	9.3 ± 1.4	9.0 ± 1.1
*p*-Value	0.65	0.04	0.56
Adjusted *p*-value	0.61	0.06	0.62
The type of restaurant you work in			
Fast food	9.3 ± 1.8	9.2 ± 1.3	8.9 ± 1.2
Traditional	9.5 ± 1.9	9.6 ± 1.3	8.9 ± 0.9
Fine dining	9.5 ± 1.6	9.3 ± 1.3	8.9 ± 1.2
Banquet kitchen	8.8 ± 1.8	9.2 ± 1.3	8.9 ± 1.0
*p*-Value	0.17	0.06	0.86
Adjusted *p*-value	0.15	0.06	0.75

One-way ANOVA test and independent sample *t*-test were used to examine the difference. Adjusted *p*-value was determined using regression analysis adjusted for gender, age, education, monthly income, nationality, year of experience in Saudi Arabia, marital status, and number of safety courses attended depending on the testing factor.

**Table 6:** Association between total knowledge, attitudes, and practices (KAP), total KAP %, poor and good groups, and food handlers’ socio-demographic characteristics.

Sociodemographic Characteristic	Total KAP	Total KAP (%)	Poor (*n* = 69) *n* (%)	Good (*n* = 320) *n* (%)
	Mean ± Std	Mean ± Std		
Gender				
Male	27.5 ± 2.7	74.5 ± 7.2	61 (88.4)	305 (95.3)
Female	27.7 ± 3.6	74.9 ± 9.7	8 (11.6)	15 (4.7)
*p*-Value	0.79	0.79	0.02	
Adjusted *p*-value	0.94	0.94		
Age				
18–28	27.5 ± 2.8	74.4 ± 7.5	31 (44.9)	144 (45.0)
29–39	27.4 ± 2.7	74.1 ± 7.3	28 (40.6)	121 (37.8)
40–49	27.8 ± 2.3	74.9 ± 6.3	9 (13.0)	40 (12.5)
50 and above	29.1 ± 2.8	78.5 ± 7.6	1 (1.4)	15 (4.7)
*p*-Value	0.13	0.13	0.66	
Adjusted *p*-value	0.15	0.15		
Marital status				
Married	27.8 ± 2.6	75.1 ± 7.1	28 (40.6)	169 (52.8)
Unmarried	27.3 ± 2.7	73.8 ± 7.4	41 (59.4)	151 (47.2)
*p*-Value	0.09	0.09	0.08	
Adjusted *p*-value	0.31	0.31		
Educational level				
Literate	28.2 ± 2.5	76.3 ± 7.0	5 (7.2)	21 (6.6)
High school or below	27.3 ± 2.6	73.9 ± 7.1	40 (58.0)	178 (55.6)
Diploma or bachelor	27.7 ± 2.7	74.9 ± 7.5	24 (34.8)	121 (37.8)
*p*-Value	0.17	0.17	0.88	
Adjusted *p*-value	0.22	0.22		
Nationality				
Asian	27.5 ± 2.5	74.5 ± 6.8	23 (33.3)	121 (37.8)
African	27.5 ± 2.5	74.3 ± 6.8	16 (23.2)	78 (24.4)
Saudi	27.9 ± 2.8	75.4 ± 7.6	6 (8.7)	59 (18.4)
Yemeni	27.2 ± 3.1	73.6 ± 8.5	20 (29.0)	49 (15.3)
Others	27.5 ± 2.9	74.5 ± 7.8	4 (5.8)	13 (4.1)
*p*-Value	0.73	0.72	0.04	
Adjusted *p*-value	0.73	0.73		
Number of years of work in Saudi Arabia				
Less than 1 year	27.0 ± 2.8	73.1 ± 7.7	25 (36.2)	74 (23.1)
1–5 years	27.7 ± 2.7	74.9 ± 7.2	22 (31.9)	116 (36.3)
6–10 years	27.6 ± 2.5	74.7 ± 6.7	9 (13.0)	59 (18.4)
More than 10 years	27.8 ± 2.8	75.2 ± 7.4	13 (18.8)	71 (22.2)
*p*-Value	0.16	0.16	0.14	
Adjusted *p*-value	0.22	0.22		
Have you ever worked outside of Saudi Arabia?				
No	27.5 ± 2.7	74.5 ± 7.4	51 (73.9)	228 (71.3)
Yes	27.6 ± 2.6	74.5 ± 7.1	18 (26.1)	92 (28.7)
*p*-Value	0.96	0.96	0.65	
Adjusted *p*-value	0.97	0.97		
Your job in the restaurant				
Head chef	27.6 ± 2.5	74.7 ± 7.0	12 (17.4)	66 (20.6)
Chef	27.5 ± 2.7	74.5 ± 7.5	57 (82.6)	254 (79.4)
*p*-Value	0.82	0.81	0.54	
Adjusted *p*-value	0.99	0.99		
Do you have a health certificate?				
No	27.5 ± 2.5	74.3 ± 6.6	23 (33.3)	100 (31.3)
Yes	27.6 ± 2.8	74.6 ± 7.6	46 (66.7)	220 (68.8)
*p*-Value	0.65	0.65	0.73	
Adjusted *p*-value	0.87	0.87		
How many food safety courses have you attended?				
None	27.6 ± 2.5	74.7 ± 6.8	12 (17.4)	75 (23.4)
One course	27.4 ± 2.6	73.9 ± 6.9	26 (37.7)	92 (28.7)
Two courses	28.0 ± 3.0	75.7 ± 8.2	13 (18.8)	69 (21.6)
More than two	27.3 ± 2.7	73.9 ± 7.4	18 (26.1)	84 (26.3)
*p*-Value	0.30	0.29	0.45	
Adjusted *p*-value	0.36	0.36		
Your monthly income				
Less than 500 SAR	27.5 ± 2.2	74.3 ± 5.9	6 (8.7)	23 (7.2)
500–1500 SAR	27.8 ± 2.5	75.2 ± 6.6	19 (27.5)	105 (32.8)
1501–2500 SAR	27.1 ± 2.6	73.2 ± 7.0	20 (29.0)	80 (25.0)
More than 2500 SAR	27.7 ± 3.0	74.8 ± 8.2	24 (34.8)	112 (35.0)
*p*-Value	0.20	0.20	0.79	
Adjusted *p*-value	0.08	0.08		
The type of restaurant you work in				
Fast food	27.4 ± 2.7	74.2 ± 7.3	36 (52.2)	144 (45.0)
Traditional	28.1 ± 2.5	75.9 ± 6.9	6 (8.7)	71 (22.2)
Fine dining	27.7 ± 2.8	74.8 ± 7.6	15 (21.7)	71 (22.2)
Banquet kitchen	26.9 ± 2.6	72.7 ± 7.0	12 (17.4)	34 (10.6)
*p*-Value	0.09	0.09	0.04	
Adjusted *p*-value	0.10	0.10		

One-way ANOVA test and independent sample *t*-test were used to examine the difference. Adjusted *p*-value was determined using regression analysis adjusted for gender, age, education, monthly income, nationality, year of experience in Saudi Arabia, marital status, and number of safety courses attended depending on the testing factor.

## Data Availability

The original contributions presented in the study are included in the article, further inquiries can be directed to the corresponding author.
